# UNION-DRIVEN PROGRESS IN WASHINGTON STATE: IMPLICATIONS FOR POLICY AND PRACTICE

**DOI:** 10.1093/geroni/igae098.1989

**Published:** 2024-12-31

**Authors:** Kezia Scales, Adam Glickman, Mariana Morante

**Affiliations:** PHI, New York, New York, United States; SEIU 775, Seattle, Washington, United States; SEIU 775, Seattle, Washington, United States

## Abstract

Recruitment and retention challenges related to direct care workers across long-term services and supports continue. Policy and practice at the federal, state, community and organizational level has the potential to improve retention, widen recruitment pipelines and create new person-centered models that attend to both the needs of workers and people who receive services and their care partners. With input from SEIU 775 leadership, this presentation will provide an overview of union reach in Washington state within the LTSS sectors, review some of the interplay between city-based living wage laws and wage improvements for direct care workers, and talk about successes in improving access and affordability of health insurance for workers and increased access to paid-time off. Panelists will ask questions related to agenda setting and co-design of solutions related to both research and policy. Policy and practice levers at the federal, state and community levels will be identified and discussed using a co-design framework.

